# The Magnitude and Determinants of Missed Opportunities for Childhood Vaccination in South Africa

**DOI:** 10.3390/vaccines8040705

**Published:** 2020-11-25

**Authors:** Duduzile Ndwandwe, Chukwudi A. Nnaji, Charles S. Wiysonge

**Affiliations:** 1 Cochrane South Africa, South African Medical Research Council, Francie Van Zijl Drive, Parow Valley, Cape Town 7501, South Africa; NNJCHU001@myuct.ac.za (C.A.N.); Charles.Wiysonge@mrc.ac.za (C.S.W.); 2 School of Public Health and Family Medicine, University of Cape Town, Anzio Road, Observatory, Cape Town 7925, South Africa; 3 Department of Global Health, Stellenbosch University, Francie Van Zijl Drive, Cape Town 7505, South Africa

**Keywords:** missed opportunities for vaccination, vaccination coverage, South Africa, demographic and health survey, low- and middle-income countries

## Abstract

Missed opportunities for vaccination (MOV) may be among the factors responsible for suboptimal vaccination coverage in South Africa. However, the magnitude and determinants of MOV in the country are not known. Thus, this study seeks to assess the prevalence and determinants of MOV in the country. South Africa is sub-divided into nine administrative provinces. We used nationally representative data from the 2016 South African Demographic and Health Survey. We considered MOV to have occurred if a child aged 12–23 months old had not taken all scheduled basic vaccine doses despite having any of the following contacts with health services: delivery in a health facility; postnatal clinic visit; receipt of vitamin A; and any child-related treatment at a health facility. Multilevel logistic regression was used to determine factors associated with MOV. The national prevalence of MOV among children aged 12–23 months was 40.1%. Children whose mothers attended facility-based antenatal care were considerably less likely to experience MOV than those whose mothers did not attend antenatal care: odds ratio (OR) 0.41, 95% confidence interval (CI) 0.19 to 0.88. Conversely, the independent predictor of an increased MOV among children was residence in either the Gauteng province (OR 2.97, 95% CI 1.29 to 6.81) or Mpumalanga province (OR 2.32, 95%CI 1.04 to 5.18); compared to residence in the Free State province. Our findings suggest a high burden of MOV among children in South Africa and that MOV may be associated with individual and contextual factors. The findings also underscore the need for further exploration of the contextual factors contributing to MOV in South Africa.

## 1. Introduction

Vaccines currently save more than three million lives from vaccine-preventable diseases every year worldwide [1]. However, vaccines can only prevent diseases if they reach the intended target populations [2]. In low- and middle-income countries (LMICs), the benefits of vaccines are not being maximised for children as routine immunisation coverage remains suboptimal [2]. Like many other LMICs, South Africa is experiencing challenges with optimising its national immunisation coverage, with a gradual decline in uptake of the third dose of diphtheria–tetanus–pertussis-containing vaccines (DTP3) from 85% in 2014 to 77% in 2019 [3].

Missed opportunities for vaccination (MOV) are said to be among the factors responsible for the suboptimal coverage [4]. MOV refer to any contact with health services by an individual who is eligible for vaccination (e.g., a child who is unvaccinated or partially vaccinated and free of contraindications to vaccination), which does not result in the person receiving the vaccine dose(s) for which they are eligible for [5]. MOV occurs in two major contexts: during visits for preventive services (e.g., vaccination) and visits for curative services (e.g., treatment for diarrhoea). In both settings, eliminating missed opportunities will increase immunisation coverage and thus prevent vaccine-preventable diseases.

Missed opportunities for childhood vaccination have been attributed to several factors at individual, community, and health-system levels [6]. There is limited evidence on the magnitude of MOV in South Africa and the factors associated with it. Jacob and Coetzee assessed the prevalence of MOV in 2015 at healthcare facilities in the city of Cape Town in the Western Cape province of South Africa, using health facility-based exit surveys of caregivers with children up to five years of age. They found the prevalence of MOV to be 4.6% [7]. This finding may not be nationally representative. Understanding the burden of MOV in South Africa will allow for MOV reduction strategies to improve vaccination coverage in the country. Assessment of various factors contributing to MOV across South Africa becomes even more crucial for mitigating the impact of the coronavirus disease 2019 pandemic on immunisation services in the country [8]. The proposed study provides novel insights into the magnitude and determinants of MOV to enable contextually tailored strategies to address these barriers. The aim of the study was to determine the prevalence of MOV in South Africa and factors associated with it.

## 2. Materials and Methods

### 2.1. Data Source

We used data from the South African Demographic and Health Survey 2016 (SADHS 2016), which was conducted from 27 June 2016 to 4 November 2016. The SADHS 2016 received ethical approval from the Human Research Ethics Committee of the South African Medical Research Council. Our study is a secondary analysis of the existing SADHS 2016 data and did not require ethical approval. The SADHS 2016 was a cross-sectional nationally representative household survey. The sampling design involved selecting and interviewing separately nationally representative probability samples of women aged 15–49 years and men aged 15–59 years based on multi-stage cluster sampling. South Africa consists of nine administrative provinces. The sample for the SADHS 2016 was designed to provide estimates of key indicators for the country, for urban and rural areas separately, and for each province. Face-to-face interviews were used to collect socioeconomic, reproductive health, prenatal and postnatal care, nutrition, vaccination, and HIV data. Vaccination information was collected from vaccination cards and mothers’ verbal reports. Interviewers asked mothers to present the vaccination cards to obtain vaccination dates. In the absence of vaccination cards, mothers were asked to recall the vaccinations that their children had received. Detailed methods and data collection procedures of the SADHS 2016 are described elsewhere [9].

### 2.2. Outcome Variable

We used the World Health Organization (WHO) definition of MOV [5] as the outcome variable for this study. MOV was defined as a binary variable that took the value of 1 if a child aged 12–23 months was not fully vaccinated despite making contact with healthcare services, and 0 otherwise. We considered a child aged 12–23 months to be fully vaccinated if they had received all recommended vaccine doses for the six WHO-recommended basic antigens in the first year of life by the time of the survey. These basic antigens include bacillus Calmette–Guérin (BCG) (one dose), polio vaccine (four doses), diphtheria–tetanus–pertussis-containing vaccines (DTP) (three doses), and measles-containing vaccines (MCV) (one dose) [10]. The South African national immunisation schedule is illustrated in Table 1 [4]. We used the report of any of the following to define contact with healthcare services: delivery in a healthcare facility; attendance at postnatal well-baby clinic; receipt of vitamin A at a healthcare facility; and recent treatment for diarrhoea, cough, or fever at a healthcare facility [11]. We adapted the WHO definition of MOV, which is based on the proportion of contacts with the health service where vaccination was due but not given, to measure the proportion of children aged 12–23 months who had missed scheduled vaccination despite at least one contact with the health service during that period.

### 2.3. Determinant Variables

We included the following determinant variables: mother’s age (15–24, 25–34, and 35 or older); mother’s education (no education, primary, and secondary or higher); household wealth index (poor, middle, and rich); mother’s marital status (unmarried and married); mother’s occupation (not working and working); sex of the child (male and female); birth order, which means the order in which the child was born (1st–3rd, 4th–6th, and 7th plus); size of the child at birth (large, average, and small); mass media access (access and no access); antenatal care (attended and never attended); and place of residence (urban, rural, and province). Variables were included in the model based on literature findings and the availability of such variables in the DHS data.

### 2.4. Statistical Analyses

We used bivariate logistic regression analyses to examine the crude association between each determinant variable and the outcome. Following this, we used multivariable logistic regression to assess which of the determinant variables are independently associated with the outcome. We applied binomial logistic regression because of the binary nature of the outcome variable. We presented measures of association as odds ratios (OR) with their 95% confidence intervals (CI). We defined statistical significance at an alpha level of 5%. We used the Bayesian information criterion to assess the goodness of fit of the model and applied variance inflation factor to test for multicollinearity. We conducted statistical analyses using Stata statistical software (Version 14).

## 3. Results

Table 2 shows the prevalence of MOV across the various determinant variables as well as the results of bivariate and multivariate logistic regression analyses.

### 3.1. Prevalence of Missed Opportunities for Vaccination

The national prevalence of MOV among children aged 12–23 months was 40.1% (Table 2). The prevalence of MOV was higher than the national average among female children (41.5%), children of 7th+ birth order (70.0%), those born of mothers who did not attend antenatal care during pregnancy (58.8%), and those whose mothers were aged 15–24 years (41.5%). Prevalence was also higher than the national average among children from poor households (43.0%), those whose mothers had only primary education (46.0%), those whose mothers had media exposure (45.4%), those born of working mothers (42.3%), those residing in rural areas (41.1%), as well as those residing in the North West (45.5%), Gauteng (50.7%), and Mpumalanga (48.6%) provinces.

### 3.2. Factors Associated with Missed Opportunities for Vaccination

In univariate analyses, the likelihood of MOV was 57% lower in children whose mothers attended antenatal care in comparison to those whose mothers did not have any antenatal care in a facility (crude OR 0.43, 95% CI 0.22 to 0.88). Children from rich homes had a 31% reduced likelihood of MOV compared to those from poor households (OR 0.69, 95% CI 0.47 to 0.99). The likelihood of MOV was more than two times higher in children residing in the Gauteng province (OR 2.60, 95% CI 1.22 to 5.58) and the Mpumalanga province (OR 2.39, 95% CI 1.17 to 4.86), compared to children staying in the Free State province.

After adjusting for covariates, the odds of MOV in children of mothers who attended antenatal care remained substantially lower than those of parents who did have antenatal care (OR 0.41, 95% CI 0.19 to 0.88). Children residing in the Gauteng province (OR 2.97, 95% CI 1.29 to 6.81) and the Mpumalanga province (OR 2.32, 95% CI 1.04 to 5.18) also remained substantially likely to have MOV compared to children living in the Free State province. However, adjusting for covariates resulted in the loss of statistical significance for the association of MOV with wealth (Table 2).

## 4. Discussion

Our findings from this cross-sectional analysis of the SADHS 2016 data show the prevalence of missed opportunities for vaccination among children in South Africa to be substantial, at 40.1%. The findings are consistent with those of a previous study of MOV in sub-Saharan African countries using a similar method based on analysis of DHS surveys, which found an average MOV prevalence of about 40% in the region [12]. However, the observed prevalence estimates are lower than those of a previous study, which assessed MOV using the WHO measure for MOV based on exit interviews following contact with a health facility and found a much higher prevalence of 77% in eligible children from Chad and 92% in Malawi [13]. We also found that maternal and geographical factors such as the province of residence are important determinants for missed opportunities for vaccination in South Africa. Our analysis shows that antenatal care by pregnant mothers increases the chances of the child not being missed for vaccination. This is in line with previous studies that demonstrated the benefits of antenatal care on uptake of child vaccination [14,15,16,17,18].

This positive relationship between antenatal care attendance and vaccination uptake can be attributed to the fact that women who visit antenatal care receive useful information on routine childhood immunisation as she is being prepared for the arrival of their new-born babies. Regular antenatal care visits also establish good relationships between pregnant women and healthcare providers, creating opportunities for health personnel to closely interact with pregnant women and can be followed up once a child is born. Furthermore, antenatal care attendance increases the likelihood of giving birth at a healthcare facility, which is significantly associated with improved child vaccination [16,17,18]. Healthcare facility delivery allows mothers to have their children vaccinated with the first dose of vaccine at birth and also obtain information on the subsequent vaccination schedule for their children [17].

Our data further show differential odds of missed opportunities for vaccination across the nine provinces in the country. South Africa is a very unequal society, with within-country disparities that are perhaps the highest in the world [19,20,21]. South African provinces have a high level of autonomy in the development and adaptation of policies, including those related to the provision of vaccination services [22]. There may therefore be wide variation across the provinces in the logistics of ensuring access to vaccination services and, perhaps, the behavioural and social drivers of vaccination [7,23,24,25,26,27]. Thus, it is essential to conduct appropriately designed research to understand the reasons for the high burden of missed opportunities for vaccination, as well as more general reasons why a substantial proportion of children are not vaccinated in South Africa [4].

A study published in 2017 by le Roux et al. reported data on a cohort of 470 sequential births in the area surrounding Zithulele Hospital in the Eastern Cape province that was followed up in their homes at 3, 6, 9, 12, and 24 months post-birth. In this study, home-based vaccination cards were used to work out the percentages of children with all immunisations at each follow-up visit. Children with all immunisations up to date were 48.6% at 3 months, 73.3% at 6 months, 83.9% at 9 months, 73.3% at 12 months, and 73.2% at 24 months [25]. This study highlights the various reasons for some of the vaccines being missed such as stock-outs (56%), lack of awareness of the immunisation schedule (16%), and lack of clinic attendance by the mother (19%) [25]. These findings are corroborated by a study in the same province in 2019 by Iwu and colleagues, who found that two-thirds of vaccination facilities had stock-outs of childhood vaccines on the day that the researchers visited the healthcare facilities [28].

This study has provided invaluable nationally representative findings on missed opportunities for vaccination in South Africa, but it has its own limitations. In instances when home-based vaccination records were not available, data on vaccination uptake and contacts with healthcare services were based on self-reports from parents. There is thus a risk of recall bias if there were systematic differences in the accuracy with which parents remembered these past experiences. However, that risk of bias is not unique to our study; it is a shortcoming inherent in cross-sectional studies, as they obtain information on potential risk factors and the outcome at the same time. In addition, our study is based on population-level data and did not assess health-facility-level determinants of missed opportunities for vaccination such as vaccine stock-outs and inconvenient vaccination times and venues [29]. Another limitation of our study is that the definition of MOV does not adequately account for the temporality of children’s contacts with health services. As such, we might have overestimated the occurrence of MOV in children who had early neonatal contacts before being eligible for all basic vaccine doses. This limitation is, however, minimal as most children had multiple contacts, including recent treatment for common illnesses such as diarrhoea, cough, or fever at a healthcare facility. Despite these shortcomings, our study has provided important findings to inform policy and future research. Based on our findings, we recommend the implementation of quality improvement strategies that can be driven by policy to address some of the contextual challenges with vaccine uptake in South Africa. Such strategies require collaborative efforts from the healthcare facilities with researchers to improve access to this most valuable tool to prevent vaccine-preventable diseases. Our study only assessed the aggregate prevalence of missed opportunities for vaccination and associated factors in children aged 12–23 months. Hence, it is important for future research efforts to assess vaccine- and dose-specific prevalence of missed opportunities for vaccination to assess whether there is variation across specific vaccines and doses, to aid a better understanding of the factors associated with missed opportunities for vaccination in South Africa.

## 5. Conclusions

Our findings suggest that missed opportunities for vaccination are prevalent in South Africa, and therefore, to improve vaccine coverage, it is important to understand and resolve factors associated with missed opportunities for vaccination. This study further highlighted the differences that exist between the different provinces and further suggests a need for a context-specific approach in addressing missed opportunities for vaccination.

## Figures and Tables

**Table 1:** South African childhood vaccination schedule (in use since 2015).

Age	Vaccine Offered
Birth	BCG, OPV (0)
6 Weeks	OPV (1), RV (1), DTaP-IPV-Hib-HepB (1), PCV (1)
10 Weeks	DTaP-IPV-HIB-HepB (2)
14 Weeks	RV (2), DTaP-IPV-Hib-HepB (3), PCV (2)
6 months	Measles (1)
9 Months	PCV (3)
12 months	Measles (2)
18 Months	DTaP-IPV-Hib-HepB (4)
6 years	Td (1)
9 years	HPV (1), HPV (2) (2 doses, 6 months apart) *
12 years	Td (2)

* BCG = bacillus Calmette–Guérin, DTaP-IPV-Hib-HepB = pentavalent vaccine (containing diphtheria, tetanus, pertussis, injectable polio, haemophilus influenzae b and hepatitis B vaccines), HPV = human papilloma virus vaccine, OPV = oral polio vaccine, PCV = pneumococcal conjugate vaccine, RV = rotavirus vaccine, Td = tetanus and reduced dose diphtheria vaccine. The HPV vaccine is given as part of the school health programme rather than the EPI-SA.

**Table 2:** Frequency and determinants of missed opportunities for vaccination in South Africa in 2016 among children aged 12–23 months.

Variables	Missed Opportunity for Vaccination		Measures of Association	
	Yes *N* (%)	No *N* (%)	Crude Odds Ratio (95% CI)	Adjusted Odds Ratio (95% CI)
All children	284 (40.11)	424 (59.89)		
Sex of child				
Female	135 (41.54)	190 (58.46)	Reference	Reference
Male	149 (38.90)	234 (61.10)	0.89 (0.66–1.21)	0.84 (0.60–1.17)
Birth order				
1st–3rd order	238 (39.40)	366 (60.60)	Reference	Reference
4th–6th order	39 (41.49)	55 (58.51)	1.09 (0.70–1.70)	0.99 (0.58–1.69)
7th + order	7 (70.00)	3 (30.00)	3.59 (0.92–14.01)	2.85 (0.47–17.43)
Birth size				
Large	69 (37.70)	144 (62.30)	Reference	Reference
Average	166 (40.19)	247 (59.81)	1.11 (0.78–1.59)	0.98 (0.68–1.44)
Small	42 (40.38)	62 (59.62)	1.12 (0.68–1.83)	1.19 (0.70–2.03)
Maternal age (years)				
15–24	107 (41.47)	151 (58.53)	Reference	Reference
25–34	131 (38.99)	205 (61.01)	0.90 (0.65–1.26)	0.79 (0.53–1.16)
35+	46 (40.35)	68 (59.65)	0.95 (0.61–1.50)	0.75 (0.42–1.34)
Maternal education				
No education	3 (42.86)	4 (57.14)	Reference	Reference
Primary	29 (46.03)	34 (53.97)	1.14 (0.23–5.50)	0.93 (0.17–5.08)
Secondary or higher	252 (39.50)	386 (60.50)	0.87 (0.19–3.92)	0.85 (0.17–4.36)
Maternal wealth index				
Poor	165 (42.97)	219 (57.03)	Reference	Reference
Middle	58 (40.00)	87 (60.00)	0.88 (0.60–1.30)	0.84 (0.53–1.31)
Rich	61 (34.08)	118 (65.92)	0.69 (0.47–0.99) *	0.64 (0.39–1.04)
Marital status				
Not married	223 (40.47)	328 (59.53)	Reference	Reference
Married	61 (38.85)	96 (61.15)	0.93 (0.65–1.34)	1.20 (0.79–0.85)
Antenatal visits during pregnancy				
No	20 (58.82)	14 (41.18)	Reference	Reference
Yes	243 (38.33)	391 (61.67)	0.43 (0.22–0.88) *	0.41 (0.19–0.88) *
Maternal occupational status				
Working	82 (42.27)	112 (57.73)	Reference	Reference
Not working	202 (39.30)	312 (60.70)	0.88 (0.63–1.24)	0.70 (0.47–1.02)
Media access				
Has no access	39 (45.35)	47 (54.65)	Reference	Reference
Has access	245 (39.39)	377 (60.61)	0.78 (0.50–1.23)	1.00 (0.59–1.72)
Residence				
Urban	140 (39.11)	218 (60.89)	Reference	Reference
Rural	144 (41.14)	206 (58.86)	1.09 (0.81–1.47)	0.99 (0.65–1.50)
Province of residence				
Free State	15 (28.30)	38 (71.70)	Reference	Reference
Eastern Cape	33 (34.74)	62 (65.26)	1.35 (0.65–2.80)	1.33 (0.58–3.05)
Gauteng	35 (50.72)	34 (49.28)	2.61 (1.22–5.58) *	2.97 (1.29–6.81) *
KwaZulu-Natal	47 (39.17)	73 (60.83)	1.63 (0.81–3.29)	1.71 (0.77–3.79)
Limpopo	38 (38.38)	61 (61.62)	1.58 (0.77–3.25)	1.52 (0.65–3.56)
Mpumalanga	51 (48.57)	54 (51.43)	2.39 (1.18–4.86) *	2.32 (1.03–5.18) *
Northern Cape	17 (31.48)	37 (68.52)	1.16 (0.51–2.67)	1.03 (0.41–2.63)
North West	35 (45.45)	42 (54.55)	2.11 (1.00–4.46) *	2.41 (1.05–5.48) *
Western Cape	13 (36.11)	23 (63.89)	1.43 (0.58–3.54)	1.71 (0.64–4.60)

* indicates *p*-value less than 0.05, showing significant findings. *N*, absolute count; %, percentage; CI, confidence interval.
